# Experimental assessments of the cross-reactivity of IgE from patients sensitised with acid-hydrolysed wheat protein in a cosmetic soap

**DOI:** 10.1186/2045-7022-5-S3-P16

**Published:** 2015-03-30

**Authors:** Shinobu Sakai, Ryosuke Nakamura, Reiko Adachi, Yuma Fukutomi, Yoshiro Saito, Tomoko Nishimaki-Mogami, Reiko Teshima

**Affiliations:** 1 National Institute of Health Sciences, Tokyo, Japan; 2 Sagamihara National Hospital, Sagamihara, Japan

## 

Hydrolysed wheat protein (HWP), which has high emulsifying property and water retentivity, is added to various foods and cosmetics to improve their texture. Recently, several studies showed that a specific HWP formula could induce IgE-mediated hypersensitivity by skin contact and/or food ingestion. In Japan, a number of HWP-caused systemic wheat allergy cases were reported, including wheat-dependent exercise-induced anaphylaxis after sensitisation to acid-hydrolysed gluten additive [Glupearl 19S^®^ (Glp19S)] in a cosmetic soap. These allergic manifestations are believed to result from deamidated peptides in high-molecular weight fractions of Glp19S. However, relationships between the properties of HWP and its allergenicity remain unknown. Here, we experimentally assessed the cross-reactivity of IgE from Glp19S-sensitised patients' sera by examining the IgE-binding capacity to various HWPs generated under different conditions and the ability to elicit mast cell activation. We prepared three different HWP types by acidic hydrolysis (0.1M HCl treatment at 100°C), alkaline hydrolysis (1M NaOH treatment at 100°C) and enzymatic hydrolysis (Neutrase^®^ digestion at 50°C) for 0.5, 1, 3, 6, 9, 12 and 24 h, respectively.

We examined the capacity of IgE binding to various acid-HWPs and native gluten using Western blotting. After 0.5-1 h of acid hydrolysis, IgE immunoblotting with a patient's serum showed smears at around 40-70-kDa proteins. However, after 3 h of acid hydrolysis, IgE binding to 40-70-kDa proteins time-dependently decreased. Regarding mast cell activation by acid-HWPs, a 0.5-h hydrolysis sample showed dramatically increased mast cell activation, whereas native gluten had little effect on it. Even after prolonged hydrolysis, acid-HWPs still retained the ability to activate mast cells, with only slightly decreased activity levels. The alkaline and enzymatic hydrolysis samples are currently under investigation to assess cross-reactivity. In conclusion, we showed that allergenic HWP had specific molecular properties given by hydrolysis. These results showed that it may be possible to reduce the risk of HWP-caused allergy by the regulation of hydrolysis procedures.

